# Ferroptosis Inhibitory Abietane Diterpenoids from *Teucrium pilosum* Discovered Using Integrated Untargeted and Targeted Mass Spectrometry Strategies

**DOI:** 10.1002/cbdv.71918

**Published:** 2026-11-14

**Authors:** Sen Hong, Yan Yang, Hanfei Liu, Xuewu Zhang, Chao Chen, Huayong Lou, Ni Zhang, Jinyu Li, Chao Sun

**Affiliations:** ^1^ State Key Laboratory of Discovery and Utilization of Functional Components in Traditional Chinese Medicine Guizhou Medical University Natural Products Research Center of Guizhou Province Guiyang People's Republic of China

**Keywords:** abietane diterpenoids, ferroptosis inhibitory, GNPS, *Teucrium pilosum*, untargeted and targeted strategy

## Abstract

An integrated untargeted and targeted screening approach, global natural product social molecular networking (GNPS) and tandem mass spectrometry, was employed for the phytochemical investigation of *Teucrium pilosum*. This led to the isolation and characterization of three novel abietane diterpenoids (teucosums A–C, **1–3**) along with **15** known analogs (**4–18**). Their structures, including absolute configurations, were unequivocally elucidated through comprehensive spectroscopic analyses (HR‐ESI‐MS, 1D/2D NMR), electronic circular dichroism (ECD), and single‐crystal x‐ray diffraction. All isolated compounds were evaluated for their inhibitory effects against RSL3‐induced ferroptosis in HT‐22 cells. Notably, compounds **1**–**4**, **6**, **7**, **9**–**11**, **13,** and **15**–**18** exhibited significant anti‐ferroptosis activities. These findings not only enrich the chemical diversity of abietane‐type diterpenoids but also highlight *T. pilosum* as a promising source for potential ferroptosis‐inhibiting agents.

## Graphical abstract

Phytochemical investigation of Teucrium pilosum yields three new abietane diterpenoids (teucosums A–C) and 15 known analogs via GNPS molecular networking. show significant anti‐ferroptosis activity in HT‐22 cells, highlighting this plant as a promising source of ferroptosis inhibitors.

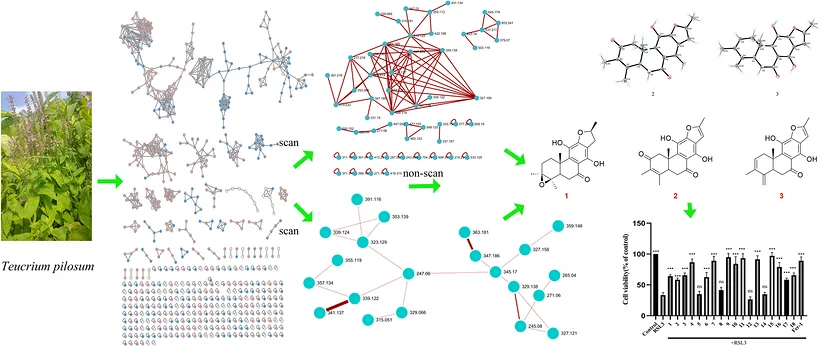

## 1 Introduction

The integration of tandem mass spectrometry–based metabolomics and chemoinformatics, natural product discovery has become one of the most efficient strategies in recent years. In particular, molecular networking based on MS^2^ data enables the clustering of compounds with similar fragmentation patterns and has thus been widely applied to link known compounds to their producing organisms as well as to discover structurally related metabolites. Currently, this method is available for online use via the open‐access Global Natural Products Social Molecular Networking (GNPS, http://gnps.ucsd.edu) platform. Using this approach, researchers have successfully identified a large number of analogues of known natural products. Recently, through studying relevant literature [1, 2, 3], our team established a strategy that employs predictive tools and characteristic mass spectrometric peaks as selection criteria, which we refer to as directed and non‐directed screening approaches.

Ferroptosis, a recently identified form of programmed cell death, is characterized by the intracellular iron‐dependent accumulation of lipid peroxides. Morphologically, biochemically, and genetically, it is distinct from the well‐known processes of apoptosis, necrosis, and autophagy. Programmed cell death plays a crucial role in human development and the maintenance of homeostasis. In recent years, increasing evidence has demonstrated that ferroptosis is closely associated with the onset and progression of neurodegenerative diseases, cerebrovascular sclerosis, and brain tumors [4]. In previous studies on the inhibition of ferroptosis in neuronal cells, it was found that the abietane‐type diterpenoids exhibited excellent ferroptosis inhibitory activity, with the best EC_50_ value of 56 nM (nanomolar) [5].

The genus *Teucrium* (Lamiaceae) consists of approximately 300 species of shrubs and herbs, mainly distributed in southwestern China. Previous phytochemical investigations of this genus have revealed that diterpenoids, particularly abietane‐ and neoclerodane‐type diterpenoids, constitute the predominant secondary metabolites [6, 7, 8, 9]. Accordingly, a comprehensive phytochemical investigation of this genus was undertaken.

Among them, *Teucrium pilosum*, traditionally used in folk medicine for the treatment of wind‐heat‐type common colds, sore throats with swelling and pain, mumps, and related inflammatory conditions, has been only limitedly investigated phytochemically, with few natural products reported to date [10]. To discover additional compounds with potential ferroptosis‐inhibitory activity, a phytochemical investigation of this species was conducted, resulting in the isolation of three new abietane diterpenoids along with **15** known compounds (Figure 1). Herein, the bioassay‐guided isolation of these metabolites and their inhibitory activities against neuronal ferroptosis are reported.

**FIGURE 1:**
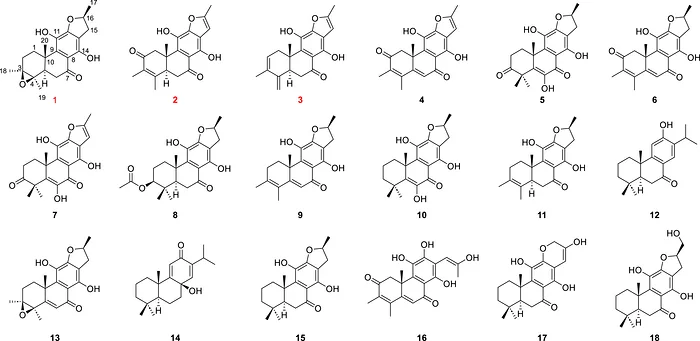
The structures of **1–18**.

## 2 Results and Discussion

### 2.1 Results

#### 2.1.1 The Screening Stage Construction and Annotation of *T. pilosum*

GNPS clustering was first performed, followed by the application of mass spectrometry prediction tools, such as SIRIUS 4 [11, 12], to determine the approximate cluster positions of the target ions. By integrating additional tools, including the GNPS LC–MS Dashboard [13], the retention‐time positions of these target ions in the LC profiles were further located. Based on their LC retention behavior, it was inferred whether the corresponding compounds were more likely to be present in normal‐phase or reversed‐phase fractions, thereby guiding the subsequent targeted isolation.

After obtaining a sufficient number of target‐related compounds, analysis of their MS/MS fragmentation patterns enabled the identification of common fragment ions (i.e., characteristic ions). These characteristic ions were then employed to screen the ethyl acetate (EA) layer MS^2^ data, followed by GNPS‐based clustering analysis. Finally, ions that remained unannotated were traced and localized through detailed chromatographic analysis.

#### 2.1.2 Untargeted Screening Stage

In the initial untargeted screening stage, liquid chromatography–mass spectrometry (LC–MS) analysis was performed in conjunction with GNPS molecular networking and in silico prediction tools such as SIRIUS 4. This integrated approach enabled the comprehensive profiling of the chemical constituents present in *T. pilosum*. The resulting molecular network revealed the presence of a large number of abietane‐type diterpenoids, as indicated by the clustering of nodes with similar MS^2^ fragmentation patterns (Figure 2). These findings highlighted the chemical diversity of abietane‐type diterpenoids in this species and provided a solid foundation for subsequent targeted isolation and structural elucidation efforts.

**FIGURE 2:**
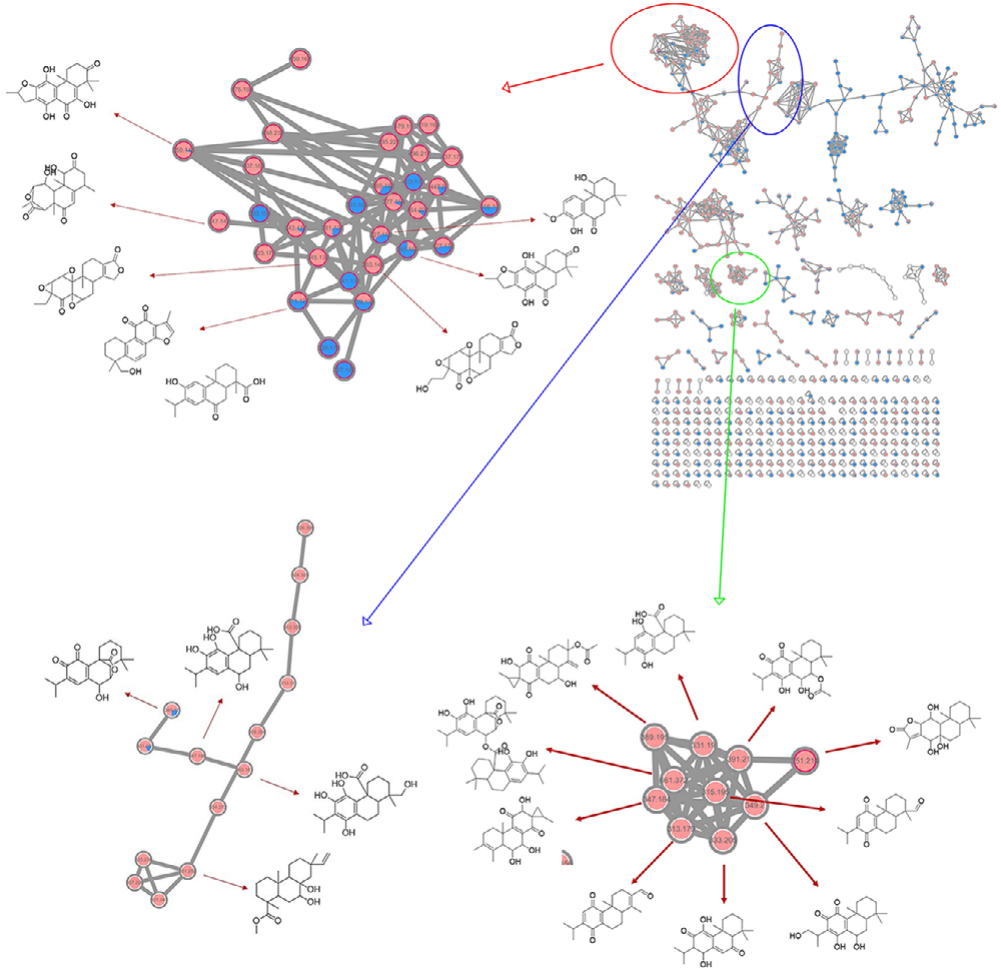
GNPS molecular networking combined with SIRIUS‐based metabolite prediction of abietane diterpenoids of *T. pilosum*.

Based on the prediction and clustering results, the precursor ion at *m/z* 359.148 exhibited the highest similarity to the target compound. Its corresponding cluster also contained several other potential targets. Consequently, this ion was further investigated. Using the GNPS LC–MS Dashboard, the feature was found predominantly in the retention time window of 15–20 min (Figure 3A), roughly corresponding to Fr. E from the normal‐phase separation (characterized by relatively high polarity). MS^2^ spectra of Fr. E were acquired, and SIRIUS4 analysis confirmed that the observed feature corresponded to the same ion. Further inspection indicated that this ion was mainly concentrated in the 19–20 min retention time range (Figure 3B). The fraction was then subjected to reversed‐phase chromatography, yielding six subfractions (Fr. E1–6). LC–MS analysis of Fr. E3 confirmed the presence of the target feature (Figure 3C). Ultimately, semi‐preparative HPLC enabled the isolation and identification of the target metabolite as teuvincenone A (**5**). Using compound **5** as a probe, a related feature was detected in the petroleum ether extract, which was subsequently assigned to compounds **4**, **6**, **9**, **10**, and **13**.

**FIGURE 3:**
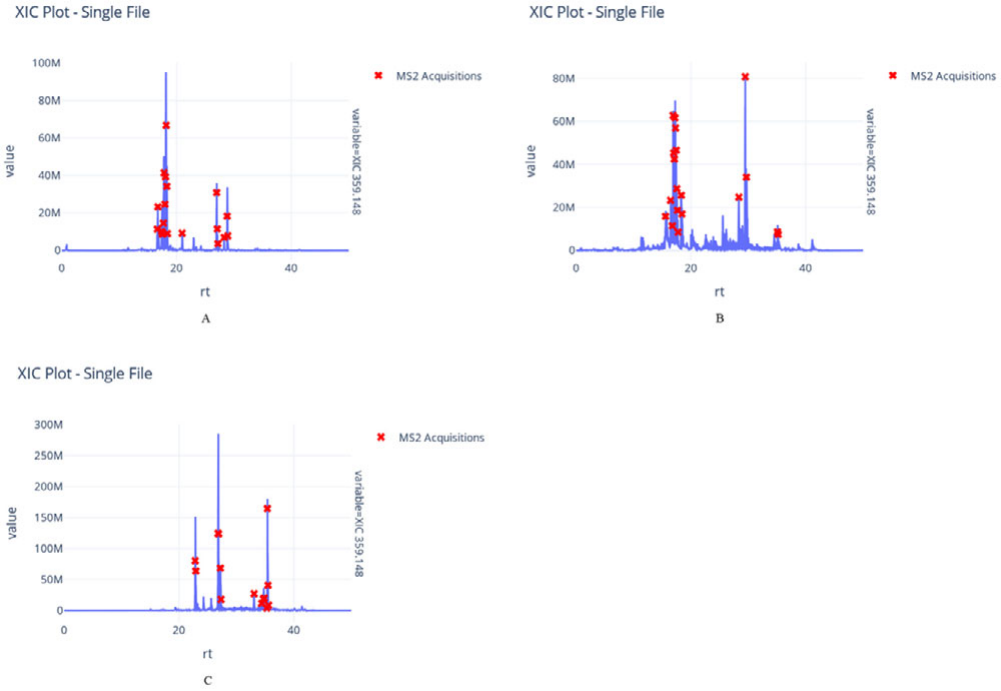
LC–MS chromatographic position of compound **5** in the PE fraction (A), fraction E of the petroleum ether layer (normal‐phase) (B), and in the E3 (reversed‐phase) (C).

#### 2.1.3 Targeted Screening Stage

MS^2^ spectra of compounds **4**−**6**, **9**, **10**, and **13** were analyzed for fragmentation patterns leading to *m/z* 243.06 or 259.09 [2, 3]. The MS^2^ data from petroleum ether (Figure 4) and EA (Figure 5) fractions were re‐screened using diagnostic ions and an in‐house code. The filtered data were then re‐imported into GNPS, which allowed the identification of additional features. Meanwhile, the original LC–MS data were processed in MZmine 3, where chromatograms were constructed using the ADAP algorithm. The retention times of the selected features were converted to the corresponding reversed‐phase fractions for targeted isolation (Table S1). As a result, several compounds were obtained. The MS^2^ spectra of three new compounds (**1**–**3**) were further analyzed, confirming the presence of the two diagnostic ions in each case. MS^2^ spectra of compounds **4**–**6**, **9**, **10**, and **13** were analyzed for fragmentation patterns leading to diagnostic ions at *m/z* 243.06 and 259.09. For *m/z* 243.06, compound **4** underwent sequential loss of two methyl groups, a carbonyl moiety, and a C_3_H_4_ unit, yielding the diagnostic ion at *m/z* 243.064. In compound **5**, stepwise losses of a methyl group, a carbonyl group, another methyl, a vinyl group, and a water molecule produced *m/z* 243.062. Compound **6** showed successive loss of a methyl radical, a carbonyl group, and another methyl group, resulting in *m/z* 243.065. These results thus confirm *m/z* 243.06 as a characteristic ion for this class of compounds (Figure S1).

**FIGURE 4:**
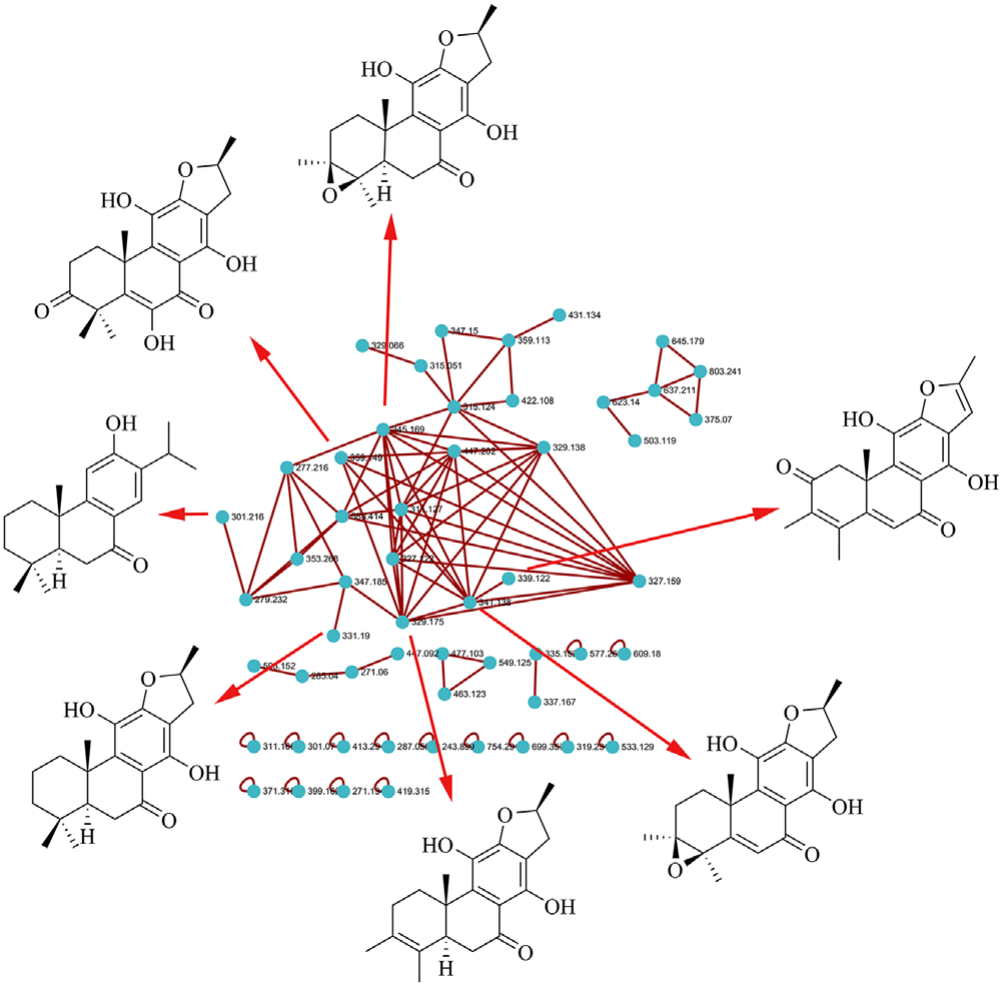
Molecular network of abietane diterpenoids from the petroleum ether (PE) fraction, filtered using characteristic ions at *m/z* 259.09 and *m/z* 243.06.

**FIGURE 5:**
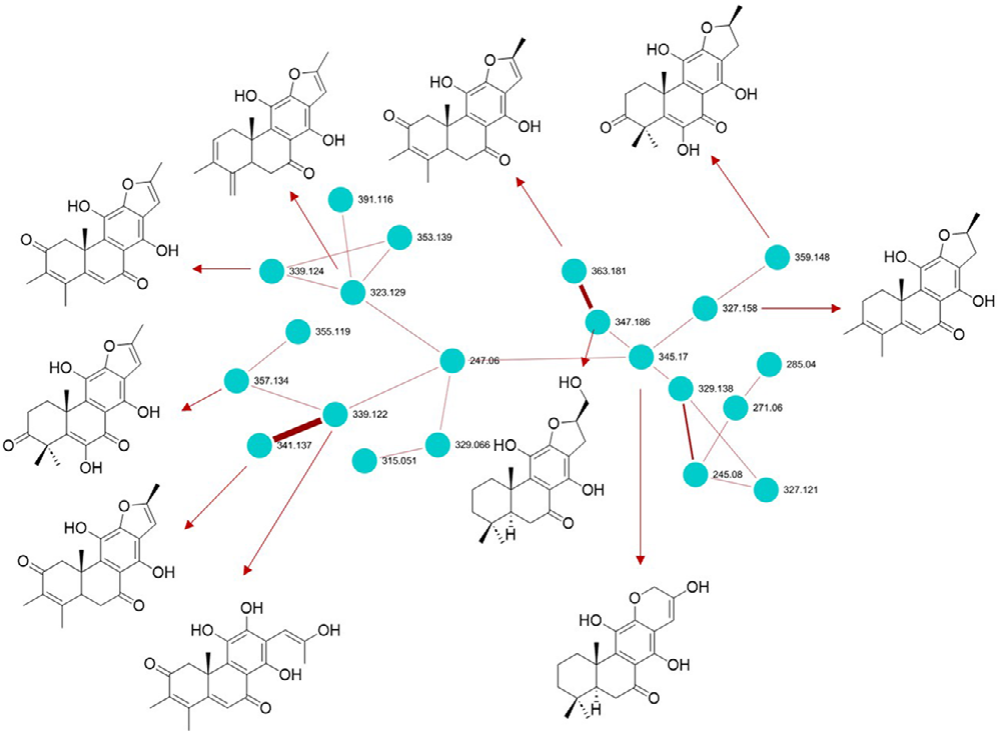
The molecular network of the EA layer filtered by characteristic ions at *m/z* 259.09 and 243.06.

For *m/z* 259.09, compounds **6**, **9**, **10**, and **13** exhibited distinct fragmentation pathways converging on this diagnostic ion. In compound **6**, protonation of the carbonyl group followed by *α*‐cleavage and elimination of a C_5_H_6_O unit yielded *m/z* 259.097. Compound **9** produced *m/z* 259.096 via loss of a C_5_H_8_ moiety. In compound **10**, dehydration was followed by five‐membered ring cleavage through an RDA reaction and subsequent loss of a propylene unit, ultimately affording *m/z* 259.096. For compound **13**, protonation of the oxygen‐containing ring and ring cleavage led to elimination of a methyl group and a C_4_H_7_O moiety, generating *m/z* 259.096. These observations demonstrate that *m/z* 259.09 is another key diagnostic ion for this structural type (Figure S2).

The MS^2^ data from petroleum ether (Figure 4) and EA (Figure 5) fractions were re‐screened using these diagnostic ions and an in‐house code. Filtered features were re‐imported into GNPS, enabling the identification of additional compounds. Original LC–MS data were also processed in MZmine 3, constructing chromatograms using the ADAP algorithm. Retention times of selected features were mapped to corresponding reversed‐phase fractions for targeted isolation (Table S1), yielding several compounds. MS^2^ spectra of three newly isolated compounds (**1**–**3**) further confirmed the presence of both diagnostic ions.

For compound **1**, initial protonation of the oxygen‐containing ring was followed by cleavage of the three‐membered ring, generating a hydroxyl group on one fragment and a carbocation on the other. Subsequent *α*‐cleavage and loss of an acetaldehyde moiety produced the ion at *m/z* 301.143, which underwent elimination of a methane unit to yield *m/z* 285.114. Finally, loss of an ethynyl group generated the diagnostic ion at *m/z* 259.098 (Figure 6A). In compound **2**, fragmentation commenced with loss of a methyl group to afford *m/z* 326.112. Sequential loss of another methyl group produced *m/z* 311.093, followed by elimination of a carbonyl moiety to give *m/z* 281.080. Final removal of a propargyl unit generated the diagnostic ion at *m/z* 243.065 (Figure 6B). For compound **3**, initial loss of a methane molecule yielded *m/z* 307.100, followed by elimination of a methyl group to generate *m/z* 292.077. Subsequent loss of a C_4_H_2_ unit afforded the diagnostic ion (Figure 6C).

**FIGURE 6:**
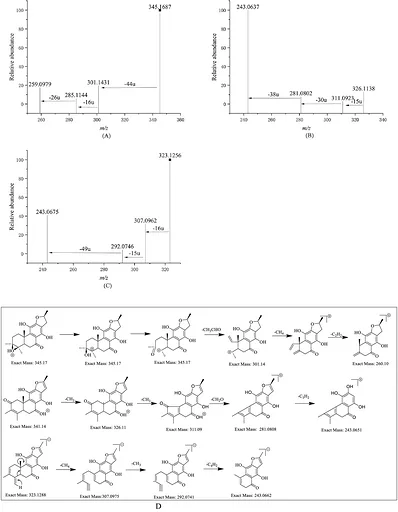
Fragmentation schematic diagrams of compounds **1–3** (A, B, C) and the proposed MS/MS fragmentation pattern of compounds **1–3** (D).

*Teucosum A* (**1**), obtained as pale white oil, was determined by HR‐ESI‐MS to have a molecular ion at *m/z* 345.1692 [M + H]^+^ (cacld. 345.1696). This corresponds to the molecular formula C_20_H_24_O_5_ and indicates nine degrees of unsaturation. The IR spectrum displayed absorption bands for hydroxyl (3745 and 3736 cm^−1^), a carbonyl group (1696 cm^−1^) and a benzene ring (1681 and 1542 cm^−1^). The ^1^H‐NMR spectrum revealed four methyl groups (*δ*_H_ 1.28, 1.34, 1.39, 1.53) and two hydroxyl group protons (*δ*_H_ 4.71, 13.22). The ^13^C‐NMR and DEPT spectra showed signals for a carbonyl carbon (*δ*_C_ 202.5), six olefinic quaternary carbons (*δ*_C_ 155.7, 155.5, 135.9, 131.6, 111.0, and 110.2), two oxygenated quaternary carbons (*δ*_C_ 62.0, 61.5), and an oxygenated methine (*δ*_C_ 83.2). Further analysis revealed one aliphatic quaternary carbon, one methine, four methylenes, and four methyls. Careful analysis of 1D NMR data of **1** indicated that its structure, a 17(15 → 16)‐abeo‐abietane, was similar to the concomitant isolated 15,16‐dihydrouncinatone (**13**) [14]. The main difference was the absence of a double bond in compound **1**, which was confirmed via one more degree of unsaturation. Furthermore, the HMBC correlations (Figure 7) of H‐18/H‐19 to C‐3 (*δ*_C_ 62.0) and C‐4 (*δ*_C_ 61.5), as well as H‐5 to C‐7 (*δ*_C_ 202.5), revealed the presence of an epoxy group between C‐3 and C‐4 and a carbonyl group at C‐7. Thus, the planer structure of **1** was determined. The relative configuration was determined by NOESY experiment (Figure 8).

**FIGURE 7:**
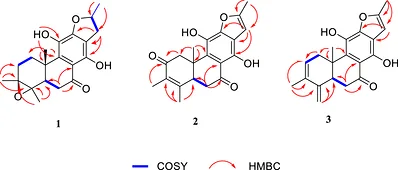
Key ^1^H‐^1^H COSY and HMBC correlations of **1–3**.

**FIGURE 8:**
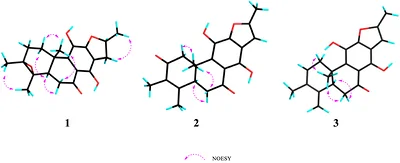
The key NOESY correlations of **1–3**.

The NOESY correlations of H_3_‐18/H_3_‐19/H‐5/H‐6*α* indicated that they were cofacial and as *α*‐oriented. Meanwhile, the NOESY cross peak H‐20/H‐6*β* suggested that H‐20 was in the *β*‐oriented; H‐1*α*/H‐5/H‐6*α* suggested that H‐5 was in the *α*‐oriented. Therefore, the absolute configuration of **1** was confirmed as 3*S*,4*R*,5*S*,10*R*,16*S* by comparing its experimental and calculated ECD curves (Figure 9).

**FIGURE 9:**
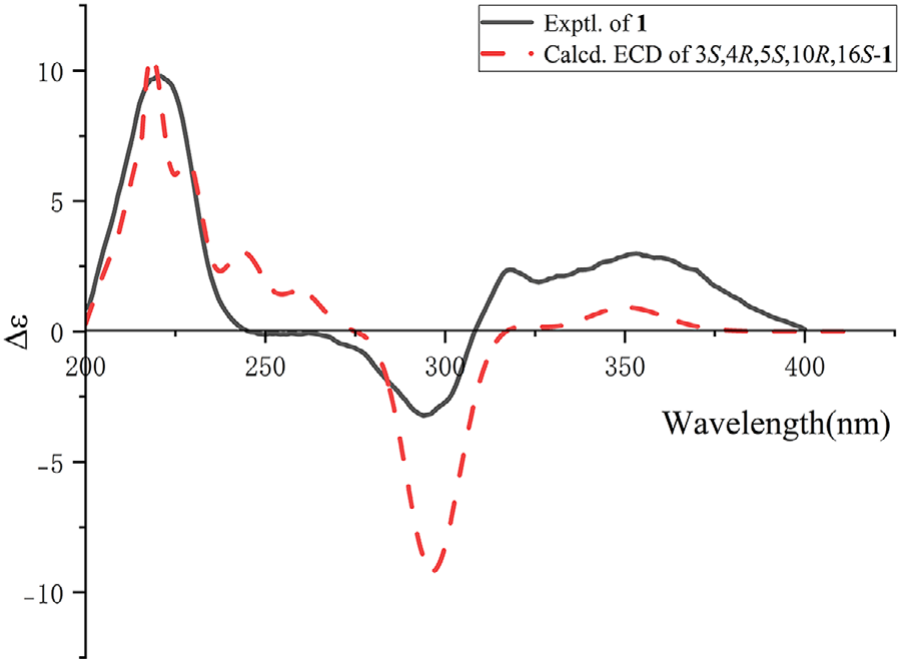
The experimental and calculated ECD spectra of **1**.

*Teucosum B* (**2**), obtained as a yellow crystal, with its molecular formula C_20_H_20_O_5_ was deduced from HR‐ESI‐MS ion peak at *m/z* 341.1382 [M + H]^+^, suggesting the presence of eleven degrees of unsaturation. Its ^1^H‐NMR spectrum indicated four methyl groups (*δ*_H_ 1.38, 1.94, 2.03, 2.50, each s, 3H), one olefinic proton signal (*δ*_H_ 6.59, s), and two hydroxyl group protons (*δ*_H_ 6.45, 13.65). The ^13^C‐NMR spectrum displayed 20 carbon signals, including two ketone carbonyl carbons (*δ*_C_ 198.2 and 202.9), ten olefinic carbons, one methine, two methylene groups, and four methyl groups (*δ*_H_ 1.38, 1.94, 2.03, 2.50, each s, 3H), one olefinic proton signal (*δ*_H_ 6.59, s), and two hydroxyl group protons (*δ*_H_ 6.45, 13.65). The ^13^C‐NMR spectrum displayed 20 carbon signals, including two ketone carbonyl carbons (*δ*_C_ 198.2 and 202.9), tenolefinic carbons, one methine, two methylene groups, and four methyl groups. The NMR data indicated that the structure of **2** was considerably similar to those of the known compound teuvincenone E (**6**) [15]. The only difference was the additional double bond between C‐15 and C‐16. This inference was supported by the degree of unsaturation and the HMBC correlations of H‐17 (*δ*_H_ 2.50) to C‐16 (*δ*_C_ 155.4) and C‐15 (*δ*_C_ 101.5). Moreover, HMBC correlation from H_3_‐18 (*δ*_H_ 1.94) to C‐2 (*δ*_C_ 198.2), C‐3 (*δ*_C_ 132.2), and C‐4 (*δ*_C_ 152.9), from H‐5 (*δ*_H_ 6.59) to C‐7 (*δ*_C_ 202.9), and C‐9 (*δ*_C_ 127.7) revealed two ketone carbonyl groups located at C‐2 and C‐7, respectively. The NOESY correlations of H_3_‐20/H‐6*β*, and H‐5/H‐6*α* demonstrated that H‐5 was *α*‐oriented, whereas H‐20 should be *β*‐oriented. Subsequently, the absolute configuration of compound **2** was unambiguously determined as 5*S*,10*S* by single‐crystal x‐ray diffraction analysis with a Flack parameter of 0.02(7) (Figure 10).

**FIGURE 10:**
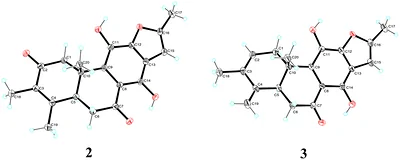
Single‐crystal x‐ray structures of **2** and **3**.

*Teucosum C* (**3**), obtained as a yellow crystal, was determined by HR‐ESI‐MS to have a molecular ion at *m/z* 323.1289 [M − H]^−^. This corresponds to the molecular formula C_20_H_20_O_4_ and indicating eleven degrees of unsaturation. The ^1^H and ^13^C NMR data (Table 1) closely resembled that of **2**, indicating that they shared the same carbon skeleton. The difference between **3** and **2** was the absence of a keto carbonyl and the presence of an additional terminal double bond in **3**. The ^1^H−^1^H COSY correlation of H_2_‐1/H_2_‐2, combined with HMBC correlations from H_3_‐18 (*δ*_H_ 1.90) to C‐2 (*δ*_C_ 125.7), C‐3 (*δ*_C_ 145.3) and C‐4 (*δ*_C_ 131.8), from H_2_‐19 (*δ*_H_ 4.91, 5.14) to C‐4 and C‐5 (43.1), identified the presence of two double bonds at ∆^2(3)^ and ∆^4(19)^ in **3**. The NOESY correlations of H_3_‐20/H‐6*β*, and H‐5/H‐6*α* indicated the H‐5 and H_3_‐20 were *α*‐ and *β*‐directed, respectively. Finally, the absolute configuration of **3** as 5*S*,10*R* was determined by single‐crystal x‐ray diffraction using Cu K*α* radiation with a Flack parameter of −0.2(2) (Figure 10).

**TABLE 1:** ^1^H‐NMR (600 MHz) data and ^13^C‐NMR (150 MHz) for compounds **1**–**3** in CDCl_3_ (*δ*_H_ and *δ*_C_ *J* in Hz).

No.	1		2		3	
	*δ*_H_ (*J* in Hz)	*δ*_C_	*δ*_H_ (*J* in Hz)	*δ*_C_	*δ*_H_ (*J* in Hz)	*δ*_C_
1*α*	1.30 (m)	28.7	2.60 (d, 12.0)	49.4	3.61 (dd, 12.0, 4.0)	39.0
1*β*	3.12 (m)		4.47 (d, 12.0)		2.40 (d, 12.0)	
2	2.01, 2.10 (m)	28.6		198.2	/	125.7
3	/	62.0	/	132.2	/	145.3
4	/	61.5	/	152.9	/	131.8
5	2.40 (dd, 15.3, 3.0 Hz)	47.0	3.27 (m)	45.3	2.92(d, 8.0)	43.1
6*α*	2.83 (t)	37.1	2.78 (dd, 12.0, 4.0)	37.5	2.74 (dd, 12.0, 4.0)	37.0
6*β*	2.84 (dd, 12.0, 8.0)		3.06 (dd, 12.0, 8.0)		2.84 (dd, 12.0, 8.0)	
7	/	202.5	/	202.9	/	204.6
8	/	111.0	/	110.5	/	117.1
9	/	135.9	/	127.7	/	130.4
10	/	37.2	/	41.0	/	38.2
11	/	131.6	/	132.2	/	132.1
12	/	155.5	/	150.2	/	149.9
13	/	110.2	/	117.8	/	117.1
14	/	155.7	/	153.7	/	152.9
15*α*	3.38 (dd, 15.3, 9.0 Hz)	34.3	6.59 (s)	101.5	/	101.6
15*β*	2.89–2.85 (m)					
16	5.15 (ddt, 13.6, 8.9, 6.4 Hz)	83.2	/	155.4	/	155.0
17	1.53 (d, 6.3 Hz)	21.9	2.50 (s)	13.8	2.47 (s)	13.9
18	1.39 (s)	20.4	1.94 (s)	11.2	1.90 (s)	19.4
19a	1.34 (s)	17.0	2.03 (s)	18.1	4.91 (s)	107.8
19b	/		/		5.14 (s)	
20	1.28 (s)	15.9	1.38 (s)	17.5	1.23 (s)	15.9
21‐OH	13.22 (s)		13.65 (s)		13.69 (s)	
22‐OH	4.71 (s)		6.46 (s)		6.60 (s)	

The remaining compounds were characterized as abietane‐type diterpenes (**4**–**18**) by extensive 1D and 2D‐NMR spectroscopy analyses and by comparison of the spectral data with those reported in literature. The known compounds were identified as teuvincenone F (**4**) [10], teuvincenone A (**5**) [15], teuvincenone E (**6**) [15], teuvincenone H (**7**) [16], 3‐acetoxylteuvincenone G(**8**) [17], uncinatone (**9**) [18], teuvincenone B(**10**) [18], 15,16‐dihydrouncinatone (**11**) [19], sugiol (**12**) [20], (3*S*,4*R*,10*R*,16*S*)‐3,4:12,16‐diepoxy‐11,14‐dihydroxy‐17(15 → 16),18(4 → 3)‐diabeo‐abieta‐5,8,11,13‐tetraene‐7‐one (**13**) [14], 8*β*,18‐dihydroxy‐9(11),13‐abietadien‐12‐one (**14**) [21], (16*S*)‐12,16‐epoxy‐11,14‐dihydroxy‐17(15 → 16)‐abeo‐abieta‐8,11,13‐trien‐7‐one (**15**) [22], crolerodendrum B (**16**) [23], 12,17‐epoxy‐11,14,16‐trihydroxy‐17(15 → 16)‐abeo‐abieta‐8,11,13,15‐tetraen‐7‐one (**17**) [24], and (16*R*)‐12,16‐epoxy‐11,14,17‐trihydroxy‐17(15 → 16)‐abeo‐8,11,13‐abietatrien‐7‐one (**18**) [10].

### 2.2 Ferroptosis Activity of Compounds **1–18** Against HT22 Cells

All isolated compounds were evaluated for their ability to inhibit ferroptosis induced by RSL3 in HT22 cells, using Fer‐1 as a positive control. Among the **18** compounds evaluated, most of the 18 compounds tested, except for **5**, **8**, **12,** and **14,** exhibited significant anti‐ferroptotic activity at a concentration of 20 µM (Figure 11). As summarized in Table 2, compounds **1**–**4**, **7**, **9**–**11**, **13**, **15**, and **18** exhibited EC_50_ values ranging from 0.255 to 2.479 µM, whereas Fer‐1 exhibited an EC_50_ of 0.030 µM. Structure‐activity relationships revealed that formation of a five‐membered oxygen‐containing ring between C‐12 and C‐13, in conjunction with a C‐16 methyl substituent, generally enhances inhibitory activity. In contrast, absence of the ring correlates with diminished activity. Moreover, even in ring‐containing analogs, introduction of carbonyl functionality at C‐2 or C‐3, or a double bond between C‐15 and C‐16, may reduce activity. As EC_50_ assays of abietane‐type diterpenoids indicated that this class of compounds exhibits notable inhibitory activity against neuronal ferroptosis. In particular, compound **10** showed the most potent activity (EC_50_ = 0.255 ± 0.077 µM), suggesting that it may be a major contributor to the observed protective effects.

**FIGURE 11:**
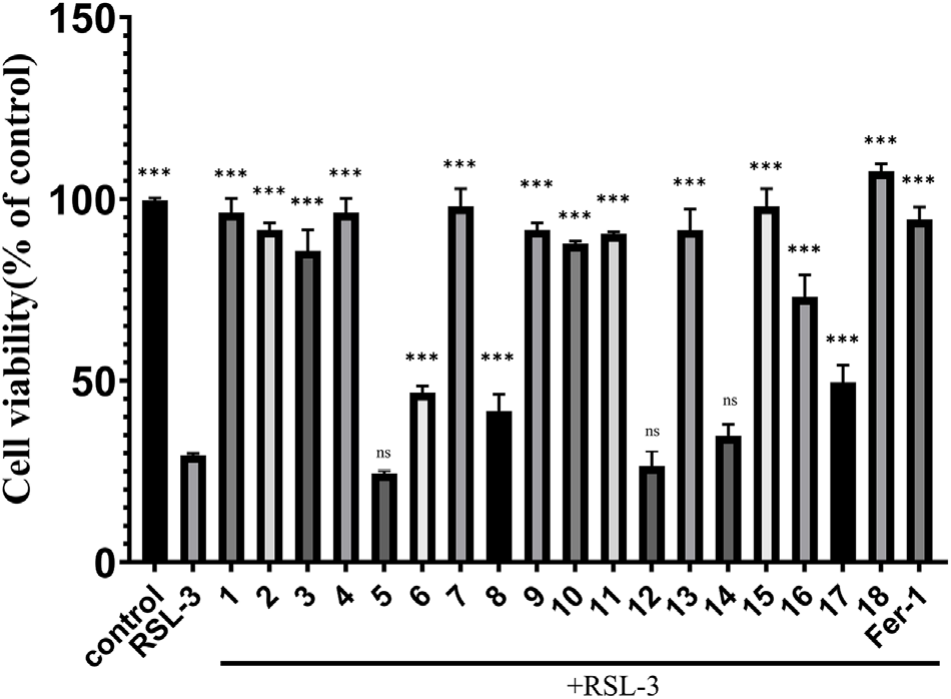
Effects of compounds **1**–**18** on RSL3‐induced HT22 cells ferroptosis. A cell viability of HT22 cells treated with 20 µM isolated compounds and 1 µM Fer‐1. Data are presented as mean ± SD (*n* = 3 technical replicates). Statistical analysis was performed using one‐way ANOVA followed by Dunnett's multiple comparisons test. *p* < 0.05, *p* < 0.01, and *p* < 0.001 indicate statistically significant differences compared with the RSL3‐treated model group.

**TABLE 2:** Effect of compounds **1**−**4, 7**, **9**‐**11**, **13**, **15**, and **18** on RSL3‐induced ferroptosis.

Compounds	EC_50_ ± SD
1	1.119 ± 0.184
2	1.079 ± 0.191
3	2.479 ± 0.379
4	1.119 ± 0.184
7	1.498 ± 0.176
9	1.079 ± 0.191
10	0.255 ± 0.077
11	0.678 ± 0.088
13	0.997 ± 0.221
15	1.498 ± 0.176
18	1.131 ± 0.082
Fer‐1	0.030 ± 0.009

## 3 Conclusions

In summary, by employing a non‐target scan and target scan strategy, three novel abietane diterpenoids with **15** known ones were isolated and characterized from the medicinal plant *T. pilosum*. The discovery of **1**–**18** not only extremely enriches the chemical diversity of abietane diterpenoids but also further demonstrates the potential of the non‐target scan and target scan strategy for rapid and efficient discovery of natural products. More encouragingly, the chemical structure confer potent inhibitory activity against RSL3‐induced ferroptosis in HT‐22 cells to compounds **1–4, 6, 7, 9–11, 13**, and **15–18**. This work provides a foundation for the further development of ferroptosis‐inhibiting therapeutics.

## 4 Experimental Section

### 4.1 Reagents and Materials

#### 4.1.1 Chemical

Analytical grade methanol and acetone were purchased from Chengdu Jinshan Chemical Reagent Co., Ltd. (Chengdu, China), and high‐performance liquid chromatography (HPLC) grade methanol and acetone from Shanghai Titan Scientific Co., Ltd. (Shanghai, China). Dulbecco's Modified Eagle Medium (DMEM) and fetal bovine serum (FBS) were purchased from Energy Chemical Co., Ltd. (Shanghai, China). DMSO and 3‐(4,5‐dimethylthiazol‐2‐yl)‐2,5‐diphenyltetrazoliumbromide (MTT) were bought from Energy Chemical Co., Ltd. (Shanghai, China). Ferrostatin‐1 (Fer‐1) was purchased from MedChemExpress (MCE) Co., Ltd.

#### 4.1.2 General Experimental Procedures

Optical rotation measurements of compounds **1**–**3** were performed at 20°C using an Autopol VI polarimeter. The UV spectrum of compound **1** was recorded on a Shimadzu UV‐2401 PC spectrophotometer, while its infrared (IR) spectrum was obtained using a Vector‐22 spectrometer. The electronic circular dichroism (ECD) spectrum of compound **1** was measured with an Applied Photophysics Chirascan circular dichroism spectrometer. The 1D and 2D NMR spectra of compounds were acquired on a Bruker Avance III 600 MHz spectrometer, with tetramethylsilane (TMS) used as the internal standard. Coupling constants are reported in hertz (Hz), and chemical shifts (*δ*) are expressed in parts per million (ppm). High‐resolution electrospray ionization mass spectrometry (HR‐ESI‐MS) data for compounds **1**–**3** were collected using an Agilent 6500 QSTAR Q Exactive Orbitrap mass spectrometer. HPLC purification was performed on an Agilent 1100 series system equipped with a Waters Sunfire C18 column (10 × 150 mm, 10 µm). For column and thin‐layer chromatography, Sephadex LH‐20 (Amersham Biosciences, Sweden), RP‐18 and MCI (Mitsubishi Chemical Industries) gel (Merck, Germany), silica gel (60–80, 100–200, 300–400 mesh; Qingdao Marine Chemical Factory, China), and silica gel GF254 TLC plates (Jiangyou Silica Gel Development Co., Ltd., Yantai, China) were employed. Chromatographic‐grade methanol and deuterated methanol were supplied by Saen Chemical Technology (Shanghai, China). All other reagents were of analytical grade, and ultrapure water was used throughout the experiments.

#### 4.1.3 Plant Material

The aerial parts of *T. pilosum* were collected in July 2022 from Anlong, Guizhou Province, China. The plant was identified by Mr. Anrui Lou, and a voucher specimen was deposited in the Natural Products Research Center of Guizhou Province (the voucher specimen: No. 20220729).

### 4.2 UPLC‐Q‐Orbitrap‐MS/MS‐Guided Isolation

#### 4.2.1 UPLC‐Q‐Orbitrap‐MS/MS

Chromatographic analysis was performed using a Thermo Scientific Dionex Ultimate 3000 UHPLC system (Sunnyvale, USA), which included an Ultimate 3000 pump, degasser, RS autosampler, and RS column compartment. The separation was conducted on a Hypersil GOLD C18 column (3.0 µm, 2.1 × 150 mm; Thermo Fisher Scientific, Waltham, MA, USA). The mobile phase consisted of water (A) and methanol (B), delivered at a flow rate of 0.2 mL/min. The injection volume was 5 µL, and the column temperature was maintained at 40°C. The gradient elution program was set as follows: 60% B (isocratic) from 0 to 3 min, linearly increasing from 60% to 100% B during 3–25 min, and held at 100% B for 25–30 min.

Mass spectrometric detection was carried out on a Q‐Exactive Orbitrap mass spectrometer (Thermo Fisher Scientific, Waltham, MA, USA) equipped with a heated electrospray ionization (HESI) source and controlled by Xcalibur 2.3 software. The HESI source parameters were as follows: spray voltage, 3.0 kV in positive mode and 2.5 kV in negative mode; capillary temperature, 320°C; sheath gas flow rate, 35 units; auxiliary gas flow rate, 10 units; and S‐lens RF level, 60. High‐resolution full‐scan mass spectra were obtained at a resolving power of 70 000 FWHM (*m/z* 200) over the range of m/z 100–1200, with an automatic gain control (AGC) target of 1 × 10^6^. Data‐dependent MS/MS acquisition was performed using higher‐energy collisional dissociation (HCD) with a normalized collision energy (NCE) of 25%, and MS/MS spectra were collected at a resolving power of 17,500 FWHM (*m/z* 200) with an AGC target of 5 × 10^4^.

#### 4.2.2 Single‐Crystal X‐Ray Diffraction Analysis

X‐ray diffraction data for suitable crystals of **2** and **3** were collected at 149.99(10) K and 112(17) K, respectively, using a XtaLAB AFC12 (RINC) Kappa single diffractometer. Structural solutions were obtained by intrinsic phasing methods implemented in SHELXT [25] and refined by full‐matrix least‐squares minimization employing SHELXL [26] within the Olex2 software package [27].

#### 4.2.3 ECD Calculation

The relative configurations of compound **1** were elucidated by NOESY data and random conformational searches using the MMFF94s force field in SYBYL‐X 2.0. The obtained conformers were optimized by TDDFT calculations at the B3LYP/6‐31G(d) level, and only stable ones without imaginary frequencies were used for ECD calculations at the B3LYP‐SCRF (PCM)/6‐31+G(d) level in methanol. ECD spectra were simulated with SpecDis v1.64 (half‐bandwidth: 0.16–0.35 eV) and UV‐corrected to generate final curves, which were compared with experimental data. All calculations were performed using Gaussian 16 [28].

### 4.3 Isolation and Purification

The dried whole plants of *T. pilosum* (51 kg) were powdered and extracted three times with 95% ethanol under reflux (2 h each). The combined extracts were concentrated under reduced pressure to remove ethanol and then suspended in hot water and successively partitioned with petroleum ether and ethyl acetate (EA). After removal of the solvents under reduced pressure, the petroleum ether fraction (1.2 kg) and the EA fraction (1.6 kg) were obtained.

The petroleum ether fraction was subjected to silica gel column chromatography (CC) using a petroleum ether–EA gradient (100:0 → 0:100) and monitored by TLC to afford six fractions (Fr. A–F). Fraction E was further separated on an ODS reversed‐phase column with a gradient of MeOH–H_2_O (60%–95%) and combined into six subfractions (Fr. E1–6) according to TLC profiles. Fr. E3 was purified by Sephadex LH‐20 CC and semi‐preparative HPLC (68% MeOH) to yield compounds **4** (12.3 mg, *t*_R_ = 23 min), **5** (8 mg, *t*_R_ = 27 min), and **7** (14 mg, *t*_R_ = 34 min). Fr. E1 was fractionated by silica gel CC with petroleum ether–EA (50:1 → 0:100) to give five subfractions (Fr. E1‐1–5). Subfraction Fr. E1‐3 was further purified by semi‐preparative HPLC (80% MeOH) to yield compounds **6** (26 mg, *t*_R_ = 16 min), and **8** (6 mg, *t*_R_ = 19 min).

Fraction C was subjected to ODS column chromatography, eluted with a gradient of MeOH–H_2_O (60–95%), and combined into seven subfractions (Fr. C1–7). Among them, Fr. C2 was further purified by Sephadex LH‐20 CC to yield crystalline compounds **9** (14 mg), **10** (18 mg), and **15** (10 mg). Fr. C3 was chromatographed on silica gel CC with petroleum ether–EA (50:1 → 0:100) to afford five subfractions (Fr. C_3_‐1–5). Subfraction C3‐2 was further purified by Sephadex LH‐20 CC and semi‐preparative HPLC to yield compounds **12** (11 mg, *t*_R_ = 18 min) and **1** (25 mg, *t*_R_ = 26 min), while subfraction C3‐3 afforded crystalline compound **13** (14 mg). Subfraction Fr.C3‐4 was purified by semi‐preparative HPLC to give compound **11** (9 mg, *t*_R_ = 23 min). Fr. C4 was also separated by silica gel CC (petroleum ether–EA, 50:1 → 0:100) to afford six subfractions (Fr. C4‐1–6). Further purification of Fr. C4‐4 by Sephadex LH‐20 CC and semi‐preparative HPLC afforded compound **14** (8 mg, *t*_R_ = 42 min).

The EA fraction was subjected to silica gel CC using a petroleum ether–EA gradient (100:0 → 0:100) and monitored by TLC to give seven fractions (Fr. A–G). Fraction F was further separated on an ODS reversed‐phase column, eluted with a gradient of MeOH–H_2_O (40–80%), to give six subfractions (Fr. F1–6). Fr. F5 was purified twice by semi‐preparative HPLC to yield compounds **2** (28 mg, *t*_R_ = 26 min) and **16** (20 mg, *t*_R_ = 28 min). Fr. F4 was purified by semi‐preparative HPLC to afford compound **3** (6 mg, *t*_R_ = 28 min). Fraction D was purified by semi‐preparative HPLC to yield five subfractions (Fr. D1–5). Fr. D4 was purified to give crystalline compound **18** (13 mg, *t*_R_ = 43 min). Further purification of Fr. D5 by semi‐preparative HPLC afforded compound **17** (10 mg, *t*_R_ = 46 min). Inspection of the ^1^H‐NMR spectra of the compounds revealed no detectable impurity signals, suggesting that their purity exceeded 95%.

#### 4.3.1 *Teucosum A* (**1**)

Pale white oil; ${\left[a\right]}_{D}^{25}$ + 20.1 (*c* 0.5, MeOH); UV (MeOH) *λ*_max_ (log *ε*): 215 (0.68) nm; CD (MeOH) *λ*_max_ (Δ*ε*): 221 (9.79) nm, IR (KBr) *υ*_max_ 3745, 3736, 1734, 1696, 1681, 1661, 1542, 1523, 1472 cm^−1^. For ^1^H‐NMR (CDCl_3_, 600 MHz) and ^13^C‐NMR (CDCl_3_, 150 MHz) spectroscopic data, see ^1^H and ^13^C NMR data, see Table 1; HR‐ESI‐MS *m/z* 345.1692 [M + H]^+^ (calcd. for C_20_H_25_O_5_, 345.1696).

#### 4.3.2 *Teucosum B* (**2**)

Pale yellow acicular crystal; ${\left[a\right]}_{D}^{25}$ + 39.4 (*c* 0.5, MeOH); mp 257°C–259°C; ^1^H and ^13^C NMR data, see Table 1. HR‐ESI‐MS *m/z* 341.1382 [M + H]^+^ (calcd. for C_20_H_21_O_5_, 341.1383).

*Crystal Data for* ***2***. Compound **2** was recrystallized from MeOH‐H_2_O (95:5) at 20°C. The crystal data of **2** (C_20_H_20_O_5_, M = 340.36 g/mol) were as follows (detailed parameters shown in Supplementary Table S2): monoclinic, space group *P*2_1_, *a* = 9.5191(2) Å, *b* = 14.2274(3) Å, *c* = 11.9375(3) Å, *α* = 90°, *β* = 94.539(2)°, *γ* = 90°, *V* = 1611.65(6) Å^3^, *Z* = 4, *T* = 149.99(10) K; *D_calc_* = 1.403 g/cm^3^; Index ranges −11 ≤ *h* ≤ 11, −17 ≤ *k* ≤ 17, −14 ≤ *l* ≤ 14; *μ* = 0.827 mm^−1^; the final *R_1_* was 0.0361 (*I* > 2*σ*(*I*)) and *wR_2_* = 0.0986 (all data). Flack parameter = 0.02(7). The crystallographic data for **2** have been deposited at the Cambridge Crystallographic Data Center (deposition number: 2496457).

#### 4.3.3 *Teucosum C* (**3**)

Pale yellow cubic crystal; ${\left[a\right]}_{D}^{25}$ +100.1 (*c* 0.5, MeOH); mp 170°C–172°C; ^1^H and ^13^C NMR data, see Table 1; HR‐ESI‐MS *m/z* 323.1289 [M − H]^−^ (calcd for C_20_H_19_O_4_, 323.1288).

*Crystal Data for* ***3***. Compound **3** was recrystallized from MeOH‐H_2_O (95:5) at 20°C. The crystal data of **3** (C_20_H_20_O_4_, M = 324.14 g/mol) were as follows (detailed parameters shown in Supplementary Table S2): tetragonal, space group *P*4_3_2_1_2, *a* = 14.4023(3) Å, *b* = 14.4023(3) Å, *c* = 15.7410(6) Å, *α* = 90°, *β* = 90°, *γ* = 90°, *V* = 3265.10(18) Å^3^, *Z* = 1, *T* = 112 (17) K; *D*_calc_ = 1.320 g/cm^3^; Index ranges −13 ≤ *h* ≤ 16, −17 ≤ *k* ≤ 9, −19 ≤ *l* ≤ 18; *μ* = 0.742 mm^−1^; the final *R_1_* was 0.0855 (*I* > 2*σ*(*I*)), 0.1080 (all data) and *wR_2_* = 0.0976 (*I* > 2*σ*(*I*)), 0.1744 (all data). Flack parameter = ‐0.2(2). The crystallographic data for **3** have been deposited at the Cambridge Crystallographic Data Center (deposition number: 2496458).

### 4.4 Cell Viability Assay

The mouse hippocampal neuronal cell line HT22 (RRID: CVCL_0321) was purchased from Procell Life Science & Technology Co., Ltd. (Wuhan, China). HT22 cells were cultured in Dulbecco's Modified Eagle Medium (DMEM, HyCyte) supplemented with 10% fetal bovine serum (HyCyte) and 1% penicillin–streptomycin (ECOTOP). The cells were maintained at 37°C in a humidified incubator with 5% CO_2_. For the cytotoxicity assay, HT22 cells were seeded into 96‐well plates at a density of 1 × 10^3^ cells per well and allowed to adhere for 24 h. The cells were then treated with RSL3 (1 µM) in the presence of the test compounds (20 µM). After 48 h of incubation, 10 µL of MTT solution (5 mg/mL) was added to each well, followed by a further 4 h incubation. The resulting formazan crystals were dissolved in 100 µL of DMSO, and the plates were gently shaken for 15 min at room temperature. The absorbance was subsequently measured at 490 nm using a microplate reader.

### 4.5 Statistical Analysis

All experiments were performed in triplicate using three technical replicates per group (*n* = 3), and the results are presented as mean ± standard deviation (SD). EC_50_ values were determined by nonlinear regression analysis using GraphPad Prism 10, with concentrations log‐transformed to base 10 prior to curve fitting. Statistical analyses were performed using GraphPad Prism 10. Differences among groups were analyzed using one‐way analysis of variance (ANOVA) followed by Dunnett's multiple comparisons test. A *p*‐value of < 0.05 was considered statistically significant.

## Author Contributions

**Sen Hong**: writing – original draft, methodology. **Yan Yang** and **Hanfei Liu**: methodology. **Xuewu Zhang**: investigation, formal analysis. **Chao Chen**: conceptualization, formal analysis. **Ni Zhang**: project administration, funding acquisition. **Jinyu Li**: supervision, writing – review and editing, funding acquisition. **Chao Sun**: project administration, supervision. All authors read and approved the final submission.

## Conflicts of Interest

The authors declare no conflicts of interest.

## Supporting information




**Supporting File 1**: cbdv71918‐sup‐0001‐SuppMat.docx

## Data Availability

The data that support the findings of this study are available in the Supporting Information of this article.
