# Supplementary material for: Ferroptosis Inhibitory Abietane Diterpenoids from Teucrium pilosum Discovered Using Integrated Untargeted and Targeted Mass Spectrometry Strategies
Source: Chem Biodivers. 2026 Nov 14;23(11):e71918. doi: 10.1002/cbdv.71918 (PMC13769032; doi:10.1002/cbdv.71918)
Supplement: Supplementary file 1 [file CBDV-23-e71918-s001.docx]

**Supporting Information**

Ferroptosis Inhibitory Abietane Diterpenoids from *Teucrium pilosum* Discovered by Integrated Untargeted and Targeted Mass Spectrometry Strategies

Sen Hong ^a^, Yan Yang ^a^, Hanfei Liu ^a^, Xuewu Zhang ^a^, Chao Chen ^a^, Huayong Lou ^a, †^, Ni Zhang ^a, *^, Jinyu Li ^a, *^, Chao Sun ^a, *^

^a^ State Key Laboratory of Discovery and Utilization of Functional Components in Traditional Chinese Medicine, Guizhou Medical University, Natural Products Research Center of Guizhou Province, Guiyang 550014, P. R. China

† Deceased. Prof. Huayong Lou passed away prior to the acceptance and publication of this manuscript. The co-authors confirm that he made a substantial scholarly contribution to the work and vouch for the absence of any conflicts of interest on his behalf

^⁎^Corresponding authors: nylzhang@163.com; [lijinyu20080@126.com](mailto:lijinyu20080@126.com); Chao_Sun2000@Hotmai.com

**Contents**

[**Figure S1.** Proposed MS/MS fragmentation pattern of compounds **4, 5, 6** to **243.065**	**3**](#_Toc202339485)

[**Figure S2.** Proposed MS/MS fragmentation pattern of compounds **6, 9, 10, 13** to **259.096**	**4**](#_Toc202339486)

[**Figure S3.** The MS/MS spectra of compound **1**	**5**](#_Toc202339486)

[**Figure S4.** The MS/MS spectra of compound **2**	**6**](#_Toc202339486)

[**Figure S5.** The MS/MS spectra of compound **3**	**7**](#_Toc202339486)

[**Figure S6.** The MS/MS spectra of compound **4**	**8**](#_Toc202339486)

[**Figure S7.** The MS/MS spectra of compound **5**	**9**](#_Toc202339486)

[**Figure S8.** The MS/MS spectra of compound **6**	**10**](#_Toc202339486)

[**Figure S9.** The MS/MS spectra of compound **9**	**11**](#_Toc202339486)

[**Figure S10.** The MS/MS spectra of compound **10**	**12**](#_Toc202339486)

[**Figure S11.** The MS/MS spectra of compound **13**	**13**](#_Toc202339486)

**Table S1**. LC–MS chromatographic position of the compound’s mass spectral peak in EA**…………………………………………………………………………………..…..14**

**Figure S12.** Code for filtering the target GNPS molecular network using characteristic ions at *m/z***.259.09** and **243.06…………………………………………………………………..16**

[**Spectra of physico-chemical properties of 1.	17**](#_Toc202339487)

[**Figure S13.** ^1^H-NMR (600 MHz) spectrum of **1** in CDCl3**.	17**](#_Toc202339488)

[**Figure S14.** ^13^C-NMR (150 MHz) spectrum of **1** in CDCl_3_**.	18**](#_Toc202339489)

[**Figure S15.** ^1^H–^1^H COSY spectrum of **1** in CDCl_3_**.	1**](#_Toc202339490)**9**

[**Figure S16.** NOESY spectrum of **1** in CDCl_3_**.	20**](#_Toc202339493)

[**Figure S17.** HSQC spectrum of **1** in CDCl_3_**.	21**](#_Toc202339491)

[**Figure S18.** HMBC spectrum of **1** in CDCl_3_	**22**](#_Toc202339492)

[**Figure S19.** UV spectrum of **1.	23**](#_Toc202339495)

[**Figure S20.** IR spectrum of **1.	24**](#_Toc202339496)

[**Figure S21.** HR-ESI-MS spectrum of **1	25**](#_Toc202339494)

[**Spectra of physico-chemical properties of 2	26**](#_Toc202339497)

[**Figure S22.** ^1^H-NMR (600 MHz) spectrum of **2** in CDCl_3_**.	26**](#_Toc202339498)

[**Figure S23.** ^13^C-NMR (150 MHz) spectrum of **2** in CDCl_3_	**27**](#_Toc202339499)

[**Figure S24.** ^1^H–^1^H COSY spectrum of **2** in CDCl_3_**.	28**](#_Toc202339500)

[**Figure S25.** NOESY spectrum of **2** in CDCl_3_**.	29**](#_Toc202339501)

[**Figure S26.** HSQC spectrum of **2** in CDCl_3_**.	30**](#_Toc202339502)

[**Figure S27.** HMBC spectrum of **2** in CDCl_3_**.	31**](#_Toc202339503)

[**Figure S28.** HR-ESI-MS spectrum of **2.	32**](#_Toc202339504)

[**Table S2** Crystal data and structure refinement for **2.	33**](#_Toc202339504)

[**Spectra of physico-chemical properties of 3	34**](#_Toc202339497)

[**Figure S29.** ^1^H-NMR (600 MHz) spectrum of **3** in CDCl_3_**.	34**](#_Toc202339508)

[**Figure S30.** ^13^C-NMR (150 MHz) spectrum of **3** in CDCl_3_**.	35**](#_Toc202339509)

[**Figure S31.** ^1^H–^1^H COSY spectrum of **3** in CDCl_3_**.	36**](#_Toc202339510)

[**Figure S32.** NOESY spectrum of **3** in CDCl_3_**.	37**](#_Toc202339511)

[**Figure S33.** HSQC spectrum of **3** in CDCl_3_**.	38**](#_Toc202339512)

[**Figure S34.** HMBC spectrum of **3** in CDCl_3_**.	39**](#_Toc202339513)

[**Figure S35.** HR-ESI-MS spectrum of **3	40**](#_Toc202339514)

[**Table S2** Crystal data and structure refinement for **3	41**](#_Toc202339504)

[**Figure S36-65.** ^1^H-NMR (600 MHz) spectrum and ^13^C-NMR (150 MHz) spectrum of **4**-**18** in CDCl_3_	**43**](#_Toc202339514)

**Fig. S1** Proposed MS/MS fragmentation pattern of compounds **4, 5** and **6** to **243.065**

**Fig. S2** Tandem mass spectrum (MS²) of compound **6**, **9**, **10** and **13** and proposed MS/MS fragmentation pattern of compounds **6, 9, 10** and **13** to 259.0964

**Fig. S3** The MS/MS spectra of compound **1**.

**Fig. S4** Tandem mass spectrum (MS²) of compound **2**

**Fig. S5** Tandem mass spectrum (MS²) of compound **3**

**Fig.S6** Tandem mass spectrum (MS²) of compound **4**

**Fig. S7** Tandem mass spectrum (MS²) of compound **5**

**Fig. S8** Tandem mass spectrum (MS²) of compound **6**

**Fig. S9** Tandem mass spectrum (MS²) of compound **9**

**Fig. S10** Tandem mass spectrum (MS²) of compound **10**

**Fig. S11** Tandem mass spectrum (MS²) of compound **13**

**Table. S1** LC–MS chromatographic position of the compound’s mass spectral peak in EA

**num = 1
for spectrum in raw_data:
 \# print (spectrum）
 params = spectrum.get('params')
 mslevel_value = params['mslevel']


 if mslevel_value=='1':
 \# print("mslevel_value is 1, storing operation")
 mgf.write([spectrum],out)
 num += 1
 elif mslevel_value =='2':
 \# print("mslevel_value is 2, checking m/z array")

 mz_array = spectrum.get('m/z array')
 mz_array_temp=mz_array
 mz_array_temp = mz_array_temp[(243 < mz_array_temp) & (mz_array_temp < 244) |
 (258 < mz_array_temp) & (mz_array_temp < 260)]

 #if(mz_array_temp=mz_array_temp[np.where(258<mz_array_temp)] and )
 \# mz_array_temp=mz_array_temp[np.where(258<mz_array_temp)]
 #mz_array_temp=mz_array_temp[np.where(260>mz_array_temp)]
 #mz_array_temp=mz_array_temp[np.where(243 < mz_array_temp)]
 #mz_array_temp=mz_array_temp[np.where(244 > mz_array_temp)]


 \# print(mz_array_temp)
 \# if the m/z array that has been filtered is not empty, so saving this m/z array
 if mz_array_temp.size>0:
 \# print("has data in the m/z array")

 \# *TODO* mgf.write([spectrum],out)
 num += 1**

**Fig. S12** Code for filtering the target GNPS molecular network using characteristic ions at *m/z* 259.09 and 243.06

**Fig. S13** The ^1^H NMR (600 MHz) of **1** in CDCl_3_

**Fig. S14** The ^13^C NMR (150 MHz) of **1** in CDCl_3_

**Fig. S15** ^1^H-^1^H COSY spectrum of **1** in CDCl_3_

**Fig. S16** NOESY spectrum of **1** in CDCl_3_

**Fig. S17** HSQC spectrum of **1** in CDCl_3_

**Fig. S18** HMBC spectrum of **1** in CDCl_3_

Fig. S19 UV spectrum of **1**

Fig. S20 IR spectrum of **1**

Fig. S21 HR-ESI-MS spectrum of **1**

**Fig. S22** The ^1^H NMR (600 MHz) of **2** in CDCl_3_

Fig. S23 The ^13^C NMR (151 MHz) of **2** in CDCl_3_

**Fig. S24** ^1^H-^1^H COSY spectrum of **2** in CDCl_3_

**Fig. S25** NOESY spectrum of **2** in CDCl_3_

**Fig. S26** HSQC spectrum of **2** in CDCl_3_

**Fig. S27** HMBC spectrum of **2** in CDCl_3_

**Fig. S28** HR-ESI-MS spectrum of **2**

| **Table S2** Crystal data and structure refinement for **2** | |
| --- | --- |
| Identification code | HS34-8 |
| Empirical formula | C_20_H_20_O_5_ |
| Formula weight | 340.36 |
| Temperature/K | 149.99(10) |
| Crystal system | monoclinic |
| Space group | P2_1_ |
| a/Å | 9.5191(2) |
| b/Å | 14.2274(3) |
| c/Å | 11.9375(3) |
| α/° | 90 |
| β/° | 94.539(2) |
| γ/° | 90 |
| Volume/Å^3^ | 1611.65(6) |
| Z | 4 |
| ρ_calc_g/cm^3^ | 1.403 |
| μ/mm^‑1^ | 0.827 |
| F(000) | 720.0 |
| Crystal size/mm^3^ | 0.15 × 0.12 × 0.09 |
| Radiation | Cu Kα (λ = 1.54184) |
| 2Θ range for data collection/° | 7.428 to 148.218 |
| Index ranges | -11 ≤ h ≤ 11, -17 ≤ k ≤ 17, -14 ≤ l ≤ 14 |
| Reflections collected | 15837 |
| Independent reflections | 6260 [R_int_ = 0.0325, R_sigma_ = 0.0370] |
| Data/restraints/parameters | 6260/1/463 |
| Goodness-of-fit on F^2^ | 1.081 |
| Final R indexes [I>=2σ (I)] | R_1_ = 0.0361, wR_2_ = 0.0976 |
| Final R indexes [all data] | R_1_ = 0.0384, wR_2_ = 0.0986 |
| Largest diff. peak/hole / e Å^-3^ | 0.17/-0.21 |
| Flack parameter | 0.02(7) |

Fig. S29 The ^1^H NMR (600 MHz) of **3** in CDCl_3_

**Fig. S30** The ^13^C NMR (151 MHz) of **3** in CDCl_3_

**Fig. S31** ^1^H-^1^H COSY spectrum of **3** in CDCl_3_

**Fig. S32** NOESY spectrum of **3** in CDCl_3_

**Fig. S33** HSQC spectrum of **3** in CDCl_3_

**Fig. S34** HMBC spectrum of **3** in CDCl_3_

**Fig. S35** HR-ESI-MS spectrum of **3**

| **Table S3** Crystal data and structure refinement for **3** | |
| --- | --- |
| Identification code | HS36-8 |
| Empirical formula | C_160_H_160_O_32_ |
| Formula weight | 2594.87 |
| Temperature/K | 112(17) |
| Crystal system | tetragonal |
| Space group | P4_3_2_1_2 |
| a/Å | 14.4023(3) |
| b/Å | 14.4023(3) |
| c/Å | 15.7410(6) |
| α/° | 90 |
| β/° | 90 |
| γ/° | 90 |
| Volume/Å^3^ | 3265.10(18) |
| Z | 1 |
| ρ_calc_g/cm^3^ | 1.320 |
| μ/mm^‑1^ | 0.742 |
| F(000) | 1376.0 |
| Crystal size/mm^3^ | 0.2 × 0.1 × 0.07 |
| Radiation | Cu Kα (λ = 1.54184) |
| 2Θ range for data collection/° | 8.682 to 148.03 |
| Index ranges | -13 ≤ h ≤ 16, -17 ≤ k ≤ 9, -19 ≤ l ≤ 18 |
| Reflections collected | 12079 |
| Independent reflections | 3237 [R_int_ = 0.0702, R_sigma_ = 0.0703] |
| Data/restraints/parameters | 3237/0/222 |
| Goodness-of-fit on F^2^ | 1.138 |
| Final R indexes [I>=2σ (I)] | R_1_ = 0.0855, wR_2_ = 0.1694 |
| Final R indexes [all data] | R_1_ = 0.1080, wR_2_ = 0.1744 |
| Largest diff. peak/hole / e Å^-3^ | 0.31/-0.35 |
| Flack parameter | -0.2(2) |

**Fig. S36** The ^1^H NMR (600 MHz) of **4** in CDCl_3_

**Fig. S37** The ^13^C NMR (151 MHz) of **4** in CDCl_3_

**Fig. S38** The ^1^H NMR (600 MHz) of **5** in CDCl_3_

**Fig. S39** The ^1^H NMR (600 MHz) of **5** in CDCl_3_

**Fig. S40** The ^1^H NMR (600 MHz) of **6** in CDCl_3_

**Fig. S41** The ^13^C NMR (151 MHz) of **6** in CDCl_3_

**Fig. S42** The ^1^H NMR (600 MHz) of **7** in CDCl_3_

**Fig. S43** The ^13^C NMR (151 MHz) of **7** in CDCl_3_

**Fig. S44** The ^1^H NMR (600 MHz) of **8** in CDCl_3_

**Fig. S45** The ^13^C NMR (151 MHz) of **8** in CDCl_3_

**Fig. S46** The ^1^H NMR (600 MHz) of **9** in CDCl_3_

**Fig. S47** The ^13^C NMR (151 MHz) of **9** in CDCl_3_

**Fig. S48** The ^1^H NMR (600 MHz) of **10** in CDCl_3_

**Fig. S49** The ^13^C NMR (151 MHz) of **10** in CDCl_3_

**Fig. S50** The ^1^H NMR (600 MHz) of **11** in CDCl_3_

**Fig. S51** The ^13^C NMR (151 MHz) of **11** in CDCl_3_

**Fig. S52** The ^1^H NMR (600 MHz) of **12** in CDCl_3_

**Fig. S53** The ^13^C NMR (151 MHz) of **12** in CDCl_3_

**Fig. S54** The ^1^H NMR (600 MHz) of **13** in CDCl_3_

**Fig. S55** The ^13^C NMR (151 MHz) of **13** in CDCl_3_

**Fig. S56** The ^1^H NMR (600 MHz) of **14** in CDCl_3_

**Fig. S57** The ^13^C NMR (151 MHz) of **14** in CDCl_3_

**Fig. S58** The ^1^H NMR (600 MHz) of **15** in CDCl_3_

**Fig. S59** The ^13^C NMR (151 MHz) of **15** in CDCl_3_

**Fig. S60** The ^1^H NMR (600 MHz) of **16** in CDCl_3_

**Fig. S61** The ^13^C NMR (151 MHz) of **16** in CDCl_3_

**Fig. S62** The ^1^H NMR (600 MHz) of **17** in CDCl_3_

**Fig. S63** The ^13^C NMR (151 MHz) of **17** in CDCl_3_

**Fig. S64** The ^1^H NMR (600 MHz) of **18** in CDCl_3_

**Fig. S65** The ^13^C NMR (151 MHz) of **18** in CDCl_3_
